# Crystal structure of ethyl­ene­dioxy­tetra­thia­fulvalene-4,5-bis­(thiol­benzoic acid) 0.25-hydrate

**DOI:** 10.1107/S2056989017011070

**Published:** 2017-08-01

**Authors:** Yuanyuan Zhang, Qiqian He, Huijie Bao, Lejia Wang, Xunwen Xiao

**Affiliations:** a Department of Material Science and Chemical Engineering, Ningbo University of Technology, 201 Fenghua Road, Ningbo 315211, People’s Republic of China

**Keywords:** crystal structure, TTF derivative, thiol­benzoate

## Abstract

In the title compound (systematic name: 4,4′-{[2-(5,6-di­hydro-[1,3]di­thiolo[4,5-*b*][1,4]dioxin-2-yl­idene)-1,3-di­thiole-4,5-di­yl]bis­(sulfanedi­yl)}di­benzoic acid 0.25-hydrate), C_22_H_14_O_6_S_6_·0.25H_2_O, the tetra­thia­fulvalene (TTF) core adopts a boat conformation, where the central S_2_C=CS_2_ plane makes dihedral angles of 31.34 (4) and 26.83 (6)°, respectively, with the peripheral S_2_C=CS_2_ and S_2_C_2_O_2_ planes. In the crystal, the benzoic acid mol­ecules are linked *via* O—H⋯O hydrogen bonds, forming inversion dimers with *R*_2_^2^(8) motifs. The dimers are linked through weak C—H⋯O hydrogen bonds into a chain structure along [-101]. The chains stack along the *a* axis through S⋯S and S⋯C short contacts, forming layers parallel to the *ac* plane.

## Abstract 2

In the crystal of the title compound, the benzoic acid mol­ecules are linked by O—H⋯O hydrogen bonds, forming inversion dimers with 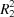
(8) motifs. The dimers are linked further *via* weak C—H⋯O hydrogen bonds, and S⋯*S* and *S*⋯C contact inter­actions into a layer structure.

## Chemical context   

Tetra­thia­fulvalene (TTF) and its derivatives have received much attention in recent years due to their unique electrical properties and synthetic versatility (Canvert *et al.*, 2009 ▸; Xiao *et al.*, 2012 ▸). Among them, bis­(ethyl­enedi­oxy)-TTF (BEDO-TTF) derivatives have afforded two-dimensional stable metallic CT complexes resulting from its self-assembling nature in partially oxidized states (Horiuchi *et al.*, 1996 ▸). Ethyl­enedi­oxy-TTF (EDO-TTF) is a noted electron-donor mol­ecule, and (EDO-TTF)_2_PF_6_ shows a metal–insulator thermal transition at near room temperature (Ota *et al.*, 2002 ▸). There are also many reports that peripheral aryl­ation of TTF could afford photochemically active organic materials. Recently, Shao’s group reported a method to introduce aryls to TTF through the sulfur atom (Sun *et al.*, 2013 ▸; Zhang *et al.*, 2015 ▸). Our group has also reported a donor mol­ecule, EDO-TTF-pyridine (Xiao *et al.*, 2012 ▸). To obtain more insight into this system, we report here the synthesis and crystal structure of the title compound.
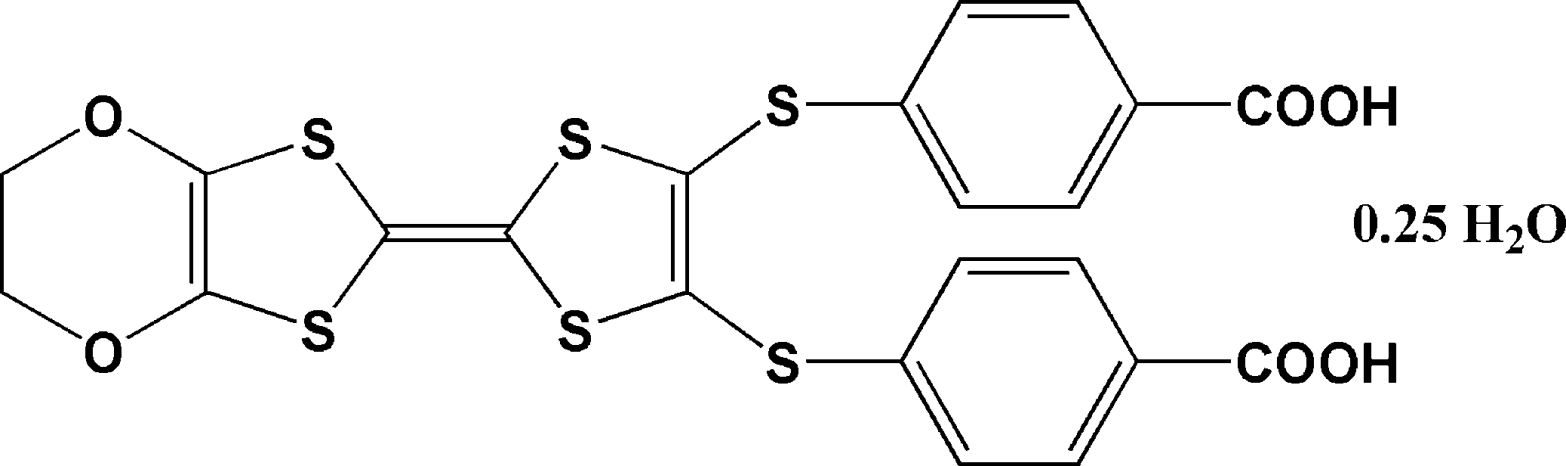


## Structural commentary   

**Figure 1:**
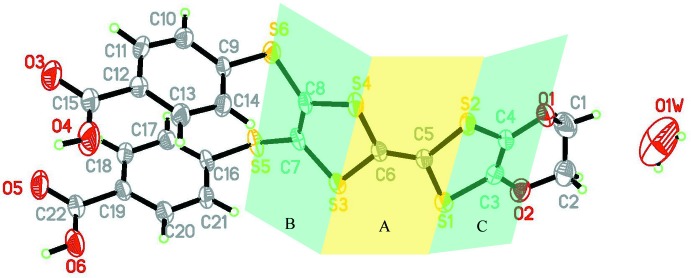
The mol­ecular structure of the title compound, showing the atom-numbering scheme. Displacement ellipsoids are drawn at the 50% probability level. *A*, *B* and *C* indicate mean planes defined by six atoms.

The asymmetric unit of the title compound contains one benzoic acid mol­ecule and a quarter mol­ecule of solvent water (Fig. 1 ▸). The TTF core adopts a boat conformation, as usually observed in neutral TTF derivatives. The central plane *A* (S1/S2/C5/C6/S3/S4) and the adjacent planes *B* (S3/S4/C7/C8/S5/S6) and *C* (S1/S2/C3/C4/O1/O2) are almost planar with r.m.s. deviations of 0.0233, 0.0274 and 0.0105 Å, respectively. The dihedral angles between planes *A* and *B* and *A* and *C* are 31.24 (4) and 26.83 (6)°, respectively. Plane *B* makes dihedral angles of 85.88 (11) and 82.03 (15)°, respectively, with the benzene C9–C14 and C16–C21 rings. These benzene rings are approximately parallel, subtending a dihedral angle of 11.82 (14)°. All bond lengths and angles in the TTF fragment are within the range of the values for a neutral TTF mol­ecule (Zhang *et al.*, 2015 ▸).

## Supra­molecular features   

**Figure 2:**
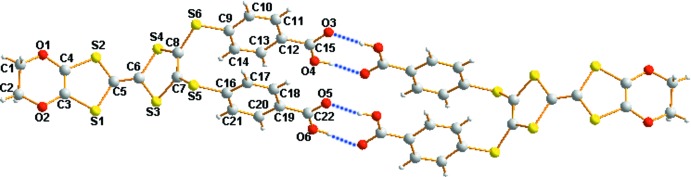
A view of the inversion dimer of the title compound with two 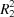
(8) hydrogen-bond motifs. O—H⋯O hydrogen bonds are shown as dotted lines.

**Figure 3:**
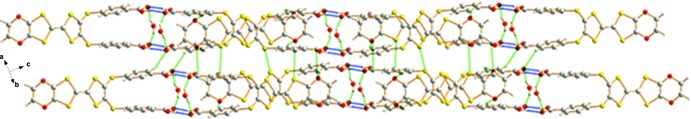
A view of the crystal packing of the title compound, showing O—H⋯O and C—H⋯O hydrogen bonds, and S⋯*S* and S⋯C inter­actions.

In the crystal, pairs of inversion-related benzoic acid mol­ecules are linked by O—H⋯O hydrogen bonds between carboxyl groups (Table 1 ▸), forming 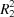 (8) hydrogen-bond motifs (Fig. 2 ▸). The water mol­ecule links two carboxyl groups in the benzoic acid mol­ecule through O—H⋯O hydrogen bonds. The dimers are linked by weak C—H⋯O hydrogen bonds into a chain structure running along [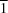
01]. The chains stack along the *a* axis *via* S⋯S and S⋯C inter­actions [S4⋯S5^iv^ = 3.420 (5) Å and S1⋯C20^v^ = 3.456 (5) Å; symmetry codes: (iv) *x* − 1, *y*, *z*; (v) −*x* + 1, −*y*, −*z* + 1], forming a layer parallel to the *ac* plane (Fig. 3 ▸).

## Database survey   

The crystal structure of 3′,4′-ethyl­enedioxo­tetra­thia­fulvolenyl-3-carb­oxy­lic acid (EDO-TTF-COOH) reported by Mézière *et al.* (2000 ▸) has a similar structure to the title compound. Both structures include O—H⋯O hydrogen bonds between carboxyl groups with 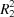 (8) ring motifs.

## Synthesis and crystallization   

**Figure 4:**
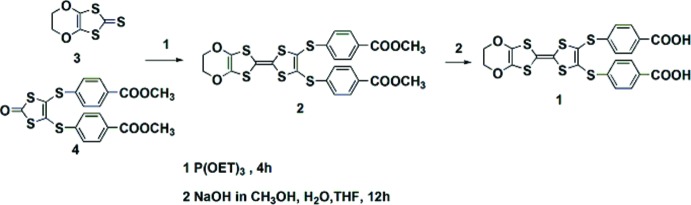
Synthesis of the title compound.

The title compound was prepared according to the reaction scheme shown in Fig. 4 ▸. 4,5-Ethyl­enedioy-1,3-di­thiole-2-thione, **3**, (systematic name: 5,6-di­hydro-[1,3]di­thiolo[4,5-*b*][1,4]dioxine-2-thione) and 4,5-bis­(thiol­methyl­benzoate)-1,3-di­thiole-2-thion, **4**, [systematic name: dimethyl 4,4′-(2-oxo-1,3-di­thiole-4,5-di­yl)bis­(sufanedi­yl)dibenzoate] were synthesized by the literature method (Sun *et al.*, 2013 ▸). Compound **2** was prepared from compounds **3** and **4** using a standard phosphite-mediated coupling procedure as follows:

Compounds **3** (193 mg 0.1 mmol) and **4** (465 mg 0.1 mmol) were mixed in tri­ethyl­phosphite (5 ml) and heated at 393 K for 6 h. P(OEt)_3_ was then removed under reduced pressure and the red residue was purified by column chromatography on silica gel (DCM) to give 310 mg of a red powder of **2** (yield = 53%). ^1^H NMR [CDCl_3_, δ (ppm), *J* (Hz)]: 8.01 (*d*, 4H, *J* = 8.5), 7.41 (*d*, 4H, *J* = 8.5), 4.28 (*s*, 4H), 3.94 (*s*, 6H).

Finally, compound **1** was obtained by hydrolysis reaction of compound **2**: A 50 ml flask was charged with compound **2** (260 mg, 0.50 mmol) under an N_2_ atmosphere. Degassed methanol (6 ml) and THF (6 ml) were added to generate a suspension. In a separate flask, sodium hydroxide (230 mg, 5.8 mmol) was dissolved in degassed water (4 ml). The sodium hydroxide solution was added to compound **2** and the reaction was heated to reflux for 8 h. The reaction was then cooled to room temperature and the volatiles were removed *in vacuo*. Hydro­chloric acid (1 mol l^−1^, 15 ml) was added to afford a maroon precipitate, which was collected by filtration and washed with water (50 ml). The product was collected and dried under high vacuum for 12 h to afford **1** as a maroon solid (179 mg, 0.35 mmol, 70% yield). ^1^H NMR [DMSO-*d*_6_, δ (ppm), *J* (Hz)]: 8.02 (*d*, 4H, *J* = 8.6),7.43 (*d*, 4H, *J* = 8.6), 4.28 (*s*, 4H). Elemental analysis calculated for C_22_H_14_O_6_S_6_: C 46.62, H 2.49%; found: C 46.67, H 2.51%. Red crystals suitable for X-ray diffraction analysis were obtained by slow evaporation of an ethyl acetate solution of the title compound. Elemental analysis calculated for C_22_H_14_O_6_S_6_·0.25H_2_O: C 46.26, H 2.56%; found: C 46.29, H 2.58%.

## Refinement   

Crystal data, data collection and structure refinement details are summarized in Table 2 ▸. Carboxyl H atoms were located in a difference-Fourier map and refined with O—H = 0.85 (2) Å, and with *U*_iso_(H) = 1.2*U*_eq_(O). H atoms bonded to C and O(water) atoms were positioned geometrically and included in the refinement in the riding-model approximation (C—H = 0.93 or 0.97 Å, and O—H = 0.85 Å) with *U*_iso_(H) = 1.2*U*_eq_(C or O). In the refinement, the occupancy of the lattice water mol­ecule was fixed at 0.25, which was estimated from the results of element analysis and gave acceptable displacement parameters for the water O atom.

## Supplementary Material

Crystal structure: contains datablock(s) I. DOI: 10.1107/S2056989017011070/is5477sup1.cif

Structure factors: contains datablock(s) I. DOI: 10.1107/S2056989017011070/is5477Isup2.hkl

Supporting information file. DOI: 10.1107/S2056989017011070/is5477Isup3.cml

CCDC reference: 1548509

Additional supporting information:  crystallographic information; 3D view; checkCIF report

## Figures and Tables

**Table 1:** Hydrogen-bond geometry (Å, °)

*D*—H⋯*A*	*D*—H	H⋯*A*	*D*⋯*A*	*D*—H⋯*A*
O4—H4⋯O5^i^	0.86 (2)	1.78 (2)	2.629 (3)	165 (5)
O6—H6⋯O3^i^	0.84 (2)	1.78 (2)	2.624 (3)	177 (4)
O1*W*—H1*WA*⋯O5^ii^	0.85	2.38	3.18 (2)	155
O1*W*—H1*WB*⋯O3^ii^	0.85	2.37	3.17 (2)	158
C13—H13*A*⋯O2^iii^	0.93	2.67	3.570 (4)	163
C20—H20*A*⋯O1^iii^	0.93	2.65	3.552 (4)	164

Symmetry codes: (i) 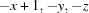; (ii) 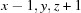; (iii) 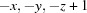.

**Table 2:** Experimental details

Crystal data	
Chemical formula	C_22_H_14_O_6_S_6_·0.25H_2_O
*M*_r_	571.19
Crystal system, space group	Triclinic, *P* 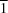
Temperature (K)	296
*a*, *b*, *c* (Å)	7.6995 (6), 9.2634 (8), 17.9198 (14)
α, β, γ (°)	90.970 (4), 92.039 (4), 110.902 (4)
*V* (Å^3^)	1192.64 (17)
*Z*	2
Radiation type	Mo *K*α
μ (mm^−1^)	0.61
Crystal size (mm)	0.32 × 0.22 × 0.16

Data collection	
Diffractometer	Bruker APEXII CCD
Absorption correction	Multi-scan (*SADABS*; Bruker, 2014 ▸)
*T*_min_, *T*_max_	0.85, 0.91
No. of measured, independent and observed [*I* > 2σ(*I*)] reflections	40263, 5496, 4411
*R*_int_	0.066
(sin θ/λ)_max_ (Å^−1^)	0.652

Refinement	
*R*[*F* ^2^ > 2σ(*F* ^2^)], *wR*(*F* ^2^), *S*	0.045, 0.119, 1.08
No. of reflections	5496
No. of parameters	322
No. of restraints	2
H-atom treatment	H atoms treated by a mixture of independent and constrained refinement
Δρ_max_, Δρ_min_ (e Å^−3^)	0.47, −0.27

Computer programs: *APEX2* and *SAINT-Plus* (Bruker, 2014 ▸), *SHELXS97* and *SHELXTL* (Sheldrick, 2008 ▸), *SHELXL2014* (Sheldrick, 2015 ▸) and *publCIF* (Westrip, 2010 ▸).

## supplementary crystallographic information

### Crystal data

**Table:**

C_22_H_14_O_6_S_6_·0.25H_2_O	*Z* = 2
*M**_r_* = 571.19	*F*(000) = 585
Triclinic, *P*1	*D*_x_ = 1.591 Mg m^−^^3^
*a* = 7.6995 (6) Å	Mo *K*α radiation, λ = 0.71073 Å
*b* = 9.2634 (8) Å	Cell parameters from 9956 reflections
*c* = 17.9198 (14) Å	θ = 2.6–27.6°
α = 90.970 (4)°	µ = 0.61 mm^−^^1^
β = 92.039 (4)°	*T* = 296 K
γ = 110.902 (4)°	Block, red
*V* = 1192.64 (17) Å^3^	0.32 × 0.22 × 0.16 mm

### Data collection

**Table:**

Bruker APEXII CCD diffractometer	4411 reflections with *I* > 2σ(*I*)
ω and φ scans	*R*_int_ = 0.066
Absorption correction: multi-scan (SADABS; Bruker, 2014)	θ_max_ = 27.6°, θ_min_ = 2.6°
*T*_min_ = 0.85, *T*_max_ = 0.91	*h* = −10→10
40263 measured reflections	*k* = −12→12
5496 independent reflections	*l* = −23→23

### Refinement

**Table:**

Refinement on *F*^2^	2 restraints
Least-squares matrix: full	Hydrogen site location: mixed
*R*[*F*^2^ > 2σ(*F*^2^)] = 0.045	H atoms treated by a mixture of independent and constrained refinement
*wR*(*F*^2^) = 0.119	*w* = 1/[σ^2^(*F*_o_^2^) + (0.0409*P*)^2^ + 1.3142*P*] where *P* = (*F*_o_^2^ + 2*F*_c_^2^)/3
*S* = 1.08	(Δ/σ)_max_ = 0.001
5496 reflections	Δρ_max_ = 0.47 e Å^−^^3^
322 parameters	Δρ_min_ = −0.27 e Å^−^^3^

### Special details

**Table:**

Geometry. All esds (except the esd in the dihedral angle between two l.s. planes) are estimated using the full covariance matrix. The cell esds are taken into account individually in the estimation of esds in distances, angles and torsion angles; correlations between esds in cell parameters are only used when they are defined by crystal symmetry. An approximate (isotropic) treatment of cell esds is used for estimating esds involving l.s. planes.

### Fractional atomic coordinates and isotropic or equivalent isotropic displacement parameters (Å^2^)

**Table:**

	*x*	*y*	*z*	*U*_iso_*/*U*_eq_	Occ. (<1)
S1	0.11044 (11)	0.09238 (9)	0.66738 (4)	0.04393 (19)	
S2	−0.09625 (9)	0.29064 (8)	0.61324 (4)	0.03501 (16)	
S3	0.32187 (10)	0.13385 (8)	0.51255 (4)	0.03623 (17)	
S4	0.12080 (9)	0.32989 (8)	0.45679 (4)	0.03457 (16)	
S5	0.66618 (9)	0.29700 (9)	0.42971 (4)	0.04079 (19)	
S6	0.42943 (10)	0.51964 (8)	0.36264 (4)	0.03834 (17)	
O1	−0.1244 (3)	0.3703 (2)	0.75549 (11)	0.0433 (5)	
O1W	−0.114 (3)	0.387 (2)	1.0200 (8)	0.189 (11)	0.25
H1WA	−0.1141	0.2992	1.0328	0.227*	0.25
H1WB	−0.2242	0.3865	1.0236	0.227*	0.25
O2	0.0741 (3)	0.1748 (3)	0.80773 (11)	0.0470 (5)	
O3	0.4480 (5)	0.2974 (3)	0.00584 (14)	0.0900 (11)	
O4	0.2328 (5)	0.0854 (3)	0.04444 (14)	0.0863 (10)	
H4	0.222 (7)	0.048 (5)	−0.0006 (14)	0.104*	
O5	0.7607 (4)	0.0500 (3)	0.08500 (12)	0.0664 (7)	
O6	0.5255 (4)	−0.1560 (3)	0.11897 (12)	0.0575 (6)	
H6	0.539 (5)	−0.200 (4)	0.0790 (14)	0.069*	
C1	−0.0452 (6)	0.3816 (4)	0.83013 (18)	0.0591 (9)	
H1A	−0.1185	0.4167	0.8640	0.071*	
H1B	0.0800	0.4579	0.8322	0.071*	
C2	−0.0387 (6)	0.2316 (4)	0.85521 (18)	0.0599 (9)	
H2A	0.0124	0.2441	0.9062	0.072*	
H2B	−0.1642	0.1559	0.8547	0.072*	
C3	0.0365 (4)	0.1960 (3)	0.73426 (15)	0.0362 (6)	
C4	−0.0543 (4)	0.2847 (3)	0.71027 (14)	0.0335 (6)	
C5	0.0641 (3)	0.1999 (3)	0.59408 (14)	0.0318 (5)	
C6	0.1508 (3)	0.2153 (3)	0.52984 (14)	0.0291 (5)	
C7	0.4361 (3)	0.2753 (3)	0.44870 (13)	0.0295 (5)	
C8	0.3442 (3)	0.3653 (3)	0.42336 (13)	0.0291 (5)	
C9	0.3906 (3)	0.4271 (3)	0.27335 (13)	0.0290 (5)	
C10	0.4906 (4)	0.5139 (3)	0.21609 (15)	0.0415 (7)	
H10A	0.5698	0.6155	0.2256	0.050*	
C11	0.4719 (5)	0.4482 (3)	0.14444 (16)	0.0472 (7)	
H11A	0.5394	0.5058	0.1062	0.057*	
C12	0.3526 (4)	0.2970 (3)	0.13006 (15)	0.0394 (6)	
C13	0.2478 (4)	0.2132 (3)	0.18680 (15)	0.0412 (7)	
H13A	0.1639	0.1134	0.1767	0.049*	
C14	0.2677 (4)	0.2776 (3)	0.25845 (14)	0.0375 (6)	
H14A	0.1986	0.2204	0.2965	0.045*	
C15	0.3430 (5)	0.2226 (4)	0.05515 (16)	0.0514 (8)	
C16	0.6443 (3)	0.2013 (3)	0.34120 (13)	0.0296 (5)	
C17	0.7745 (4)	0.2746 (3)	0.28949 (15)	0.0387 (6)	
H17A	0.8628	0.3721	0.3008	0.046*	
C18	0.7729 (4)	0.2022 (3)	0.22095 (16)	0.0419 (7)	
H18A	0.8597	0.2515	0.1862	0.050*	
C19	0.6421 (4)	0.0565 (3)	0.20418 (14)	0.0330 (5)	
C20	0.5125 (4)	−0.0176 (3)	0.25668 (15)	0.0354 (6)	
H20A	0.4252	−0.1158	0.2458	0.042*	
C21	0.5142 (3)	0.0553 (3)	0.32484 (14)	0.0340 (6)	
H21A	0.4278	0.0061	0.3598	0.041*	
C22	0.6426 (4)	−0.0211 (3)	0.13115 (15)	0.0407 (6)	

### Atomic displacement parameters (Å^2^)

**Table:**

	*U*^11^	*U*^22^	*U*^33^	*U*^12^	*U*^13^	*U*^23^
S1	0.0568 (4)	0.0448 (4)	0.0426 (4)	0.0312 (4)	0.0196 (3)	0.0122 (3)
S2	0.0351 (3)	0.0449 (4)	0.0316 (3)	0.0212 (3)	0.0112 (3)	0.0041 (3)
S3	0.0419 (4)	0.0402 (4)	0.0346 (3)	0.0238 (3)	0.0106 (3)	−0.0010 (3)
S4	0.0305 (3)	0.0527 (4)	0.0268 (3)	0.0221 (3)	0.0060 (2)	0.0005 (3)
S5	0.0269 (3)	0.0621 (5)	0.0336 (4)	0.0172 (3)	0.0012 (3)	−0.0215 (3)
S6	0.0484 (4)	0.0341 (3)	0.0279 (3)	0.0087 (3)	0.0086 (3)	−0.0067 (3)
O1	0.0529 (12)	0.0482 (12)	0.0359 (10)	0.0260 (10)	0.0124 (9)	−0.0025 (9)
O1W	0.25 (2)	0.169 (18)	0.059 (9)	−0.035 (16)	0.038 (12)	−0.041 (10)
O2	0.0530 (12)	0.0575 (13)	0.0358 (11)	0.0253 (10)	0.0093 (9)	0.0118 (9)
O3	0.165 (3)	0.0509 (15)	0.0402 (13)	0.0183 (17)	0.0469 (17)	−0.0017 (11)
O4	0.134 (3)	0.0605 (16)	0.0378 (14)	0.0012 (16)	0.0300 (15)	−0.0190 (12)
O5	0.0908 (18)	0.0584 (14)	0.0373 (12)	0.0089 (13)	0.0296 (12)	−0.0078 (10)
O6	0.0739 (16)	0.0509 (13)	0.0378 (12)	0.0099 (12)	0.0164 (11)	−0.0167 (10)
C1	0.079 (2)	0.062 (2)	0.0426 (18)	0.0330 (19)	0.0057 (16)	−0.0096 (15)
C2	0.080 (2)	0.070 (2)	0.0342 (16)	0.031 (2)	0.0124 (16)	0.0041 (15)
C3	0.0368 (14)	0.0379 (14)	0.0349 (14)	0.0133 (11)	0.0098 (11)	0.0064 (11)
C4	0.0340 (13)	0.0358 (13)	0.0325 (13)	0.0136 (11)	0.0119 (10)	0.0013 (11)
C5	0.0299 (12)	0.0326 (13)	0.0347 (13)	0.0126 (10)	0.0096 (10)	0.0013 (10)
C6	0.0269 (11)	0.0324 (13)	0.0293 (12)	0.0119 (10)	0.0048 (9)	−0.0027 (10)
C7	0.0245 (11)	0.0384 (13)	0.0262 (12)	0.0125 (10)	0.0030 (9)	−0.0115 (10)
C8	0.0293 (12)	0.0380 (13)	0.0196 (11)	0.0118 (10)	0.0043 (9)	−0.0084 (9)
C9	0.0338 (12)	0.0339 (13)	0.0230 (12)	0.0159 (10)	0.0078 (9)	−0.0014 (9)
C10	0.0545 (17)	0.0317 (14)	0.0334 (14)	0.0084 (12)	0.0131 (12)	−0.0012 (11)
C11	0.073 (2)	0.0389 (15)	0.0278 (14)	0.0164 (14)	0.0216 (14)	0.0056 (11)
C12	0.0598 (18)	0.0361 (14)	0.0244 (13)	0.0191 (13)	0.0090 (12)	−0.0002 (10)
C13	0.0513 (16)	0.0346 (14)	0.0305 (14)	0.0062 (12)	0.0087 (12)	−0.0059 (11)
C14	0.0418 (14)	0.0380 (14)	0.0272 (13)	0.0064 (12)	0.0120 (11)	−0.0017 (11)
C15	0.084 (2)	0.0416 (17)	0.0279 (14)	0.0212 (16)	0.0164 (15)	−0.0029 (12)
C16	0.0273 (11)	0.0394 (14)	0.0265 (12)	0.0173 (10)	0.0042 (9)	−0.0063 (10)
C17	0.0450 (15)	0.0324 (13)	0.0359 (14)	0.0097 (12)	0.0112 (12)	−0.0042 (11)
C18	0.0517 (17)	0.0382 (15)	0.0339 (14)	0.0122 (13)	0.0184 (12)	0.0015 (11)
C19	0.0401 (14)	0.0378 (14)	0.0255 (12)	0.0190 (11)	0.0065 (10)	−0.0040 (10)
C20	0.0331 (13)	0.0381 (14)	0.0329 (14)	0.0104 (11)	0.0047 (10)	−0.0073 (11)
C21	0.0302 (12)	0.0428 (15)	0.0289 (13)	0.0124 (11)	0.0103 (10)	−0.0042 (11)
C22	0.0544 (17)	0.0431 (16)	0.0271 (13)	0.0200 (13)	0.0090 (12)	−0.0040 (11)

### Geometric parameters (Å, º)

**Table:**

S1—C3	1.755 (3)	C2—H2A	0.9700
S1—C5	1.763 (3)	C2—H2B	0.9700
S2—C4	1.762 (3)	C3—C4	1.323 (4)
S2—C5	1.763 (3)	C5—C6	1.336 (3)
S3—C7	1.761 (3)	C7—C8	1.346 (4)
S3—C6	1.769 (2)	C9—C14	1.386 (4)
S4—C6	1.759 (3)	C9—C10	1.391 (3)
S4—C8	1.762 (2)	C10—C11	1.392 (4)
S5—C7	1.757 (2)	C10—H10A	0.9300
S5—C16	1.777 (2)	C11—C12	1.387 (4)
S6—C8	1.759 (3)	C11—H11A	0.9300
S6—C9	1.767 (2)	C12—C13	1.388 (4)
O1—C4	1.375 (3)	C12—C15	1.486 (4)
O1—C1	1.436 (4)	C13—C14	1.387 (4)
O1W—H1WA	0.8522	C13—H13A	0.9300
O1W—H1WB	0.8483	C14—H14A	0.9300
O2—C3	1.371 (3)	C16—C21	1.385 (4)
O2—C2	1.455 (4)	C16—C17	1.388 (4)
O3—C15	1.260 (4)	C17—C18	1.387 (4)
O4—C15	1.258 (4)	C17—H17A	0.9300
O4—H4	0.863 (19)	C18—C19	1.387 (4)
O5—C22	1.261 (3)	C18—H18A	0.9300
O6—C22	1.263 (4)	C19—C20	1.397 (4)
O6—H6	0.843 (18)	C19—C22	1.483 (3)
C1—C2	1.485 (5)	C20—C21	1.383 (3)
C1—H1A	0.9700	C20—H20A	0.9300
C1—H1B	0.9700	C21—H21A	0.9300
			
C3—S1—C5	91.86 (12)	C14—C9—S6	123.81 (19)
C4—S2—C5	91.46 (12)	C10—C9—S6	116.2 (2)
C7—S3—C6	93.69 (12)	C9—C10—C11	119.9 (3)
C6—S4—C8	93.76 (12)	C9—C10—H10A	120.1
C7—S5—C16	103.60 (11)	C11—C10—H10A	120.1
C8—S6—C9	103.42 (11)	C12—C11—C10	120.1 (2)
C4—O1—C1	110.0 (2)	C12—C11—H11A	120.0
H1WA—O1W—H1WB	107.7	C10—C11—H11A	120.0
C3—O2—C2	109.7 (2)	C11—C12—C13	119.8 (2)
C15—O4—H4	116 (3)	C11—C12—C15	120.2 (3)
C22—O6—H6	115 (3)	C13—C12—C15	120.0 (3)
O1—C1—C2	112.2 (3)	C14—C13—C12	120.3 (3)
O1—C1—H1A	109.2	C14—C13—H13A	119.8
C2—C1—H1A	109.2	C12—C13—H13A	119.8
O1—C1—H1B	109.2	C9—C14—C13	119.9 (2)
C2—C1—H1B	109.2	C9—C14—H14A	120.0
H1A—C1—H1B	107.9	C13—C14—H14A	120.0
O2—C2—C1	111.8 (3)	O4—C15—O3	122.8 (3)
O2—C2—H2A	109.2	O4—C15—C12	118.2 (3)
C1—C2—H2A	109.2	O3—C15—C12	119.0 (3)
O2—C2—H2B	109.2	C21—C16—C17	120.1 (2)
C1—C2—H2B	109.2	C21—C16—S5	122.88 (19)
H2A—C2—H2B	107.9	C17—C16—S5	116.8 (2)
C4—C3—O2	125.3 (3)	C18—C17—C16	119.9 (2)
C4—C3—S1	118.0 (2)	C18—C17—H17A	120.0
O2—C3—S1	116.7 (2)	C16—C17—H17A	120.0
C3—C4—O1	125.0 (2)	C19—C18—C17	120.1 (2)
C3—C4—S2	118.2 (2)	C19—C18—H18A	120.0
O1—C4—S2	116.75 (19)	C17—C18—H18A	120.0
C6—C5—S2	123.0 (2)	C18—C19—C20	119.8 (2)
C6—C5—S1	121.6 (2)	C18—C19—C22	119.8 (2)
S2—C5—S1	115.37 (14)	C20—C19—C22	120.4 (2)
C5—C6—S4	123.8 (2)	C21—C20—C19	119.9 (2)
C5—C6—S3	123.1 (2)	C21—C20—H20A	120.1
S4—C6—S3	112.94 (13)	C19—C20—H20A	120.1
C8—C7—S5	125.7 (2)	C20—C21—C16	120.2 (2)
C8—C7—S3	116.87 (18)	C20—C21—H21A	119.9
S5—C7—S3	117.18 (15)	C16—C21—H21A	119.9
C7—C8—S6	126.18 (19)	O5—C22—O6	123.6 (3)
C7—C8—S4	117.00 (19)	O5—C22—C19	118.5 (3)
S6—C8—S4	116.78 (15)	O6—C22—C19	118.0 (2)
C14—C9—C10	120.0 (2)		
			
C4—O1—C1—C2	−43.5 (4)	C9—S6—C8—S4	−100.45 (15)
C3—O2—C2—C1	−42.7 (4)	C6—S4—C8—C7	14.3 (2)
O1—C1—C2—O2	60.5 (4)	C6—S4—C8—S6	−163.43 (14)
C2—O2—C3—C4	14.2 (4)	C8—S6—C9—C14	18.4 (3)
C2—O2—C3—S1	−164.1 (2)	C8—S6—C9—C10	−162.2 (2)
C5—S1—C3—C4	12.6 (2)	C14—C9—C10—C11	−2.3 (4)
C5—S1—C3—O2	−169.0 (2)	S6—C9—C10—C11	178.3 (2)
O2—C3—C4—O1	0.4 (4)	C9—C10—C11—C12	0.5 (5)
S1—C3—C4—O1	178.6 (2)	C10—C11—C12—C13	2.0 (5)
O2—C3—C4—S2	−177.6 (2)	C10—C11—C12—C15	−175.3 (3)
S1—C3—C4—S2	0.7 (3)	C11—C12—C13—C14	−2.8 (5)
C1—O1—C4—C3	14.8 (4)	C15—C12—C13—C14	174.6 (3)
C1—O1—C4—S2	−167.3 (2)	C10—C9—C14—C13	1.5 (4)
C5—S2—C4—C3	−13.6 (2)	S6—C9—C14—C13	−179.1 (2)
C5—S2—C4—O1	168.3 (2)	C12—C13—C14—C9	1.0 (5)
C4—S2—C5—C6	−155.6 (2)	C11—C12—C15—O4	−179.9 (4)
C4—S2—C5—S1	22.02 (16)	C13—C12—C15—O4	2.7 (5)
C3—S1—C5—C6	155.8 (2)	C11—C12—C15—O3	1.9 (5)
C3—S1—C5—S2	−21.84 (17)	C13—C12—C15—O3	−175.5 (3)
S2—C5—C6—S4	1.2 (3)	C7—S5—C16—C21	−47.9 (2)
S1—C5—C6—S4	−176.19 (14)	C7—S5—C16—C17	137.0 (2)
S2—C5—C6—S3	176.14 (14)	C21—C16—C17—C18	0.8 (4)
S1—C5—C6—S3	−1.3 (3)	S5—C16—C17—C18	176.0 (2)
C8—S4—C6—C5	152.2 (2)	C16—C17—C18—C19	−0.3 (5)
C8—S4—C6—S3	−23.12 (15)	C17—C18—C19—C20	−0.3 (4)
C7—S3—C6—C5	−152.4 (2)	C17—C18—C19—C22	−179.2 (3)
C7—S3—C6—S4	23.01 (15)	C18—C19—C20—C21	0.5 (4)
C16—S5—C7—C8	−83.1 (2)	C22—C19—C20—C21	179.5 (3)
C16—S5—C7—S3	102.52 (15)	C19—C20—C21—C16	−0.1 (4)
C6—S3—C7—C8	−13.8 (2)	C17—C16—C21—C20	−0.6 (4)
C6—S3—C7—S5	161.13 (14)	S5—C16—C21—C20	−175.5 (2)
S5—C7—C8—S6	2.8 (3)	C18—C19—C22—O5	−1.7 (4)
S3—C7—C8—S6	177.20 (13)	C20—C19—C22—O5	179.4 (3)
S5—C7—C8—S4	−174.74 (13)	C18—C19—C22—O6	177.6 (3)
S3—C7—C8—S4	−0.3 (3)	C20—C19—C22—O6	−1.4 (4)
C9—S6—C8—C7	82.0 (2)		

### Hydrogen-bond geometry (Å, º)

**Table:**

*D*—H···*A*	*D*—H	H···*A*	*D*···*A*	*D*—H···*A*
O4—H4···O5^i^	0.86 (2)	1.78 (2)	2.629 (3)	165 (5)
O6—H6···O3^i^	0.84 (2)	1.78 (2)	2.624 (3)	177 (4)
O1*W*—H1*WA*···O5^ii^	0.85	2.38	3.18 (2)	155
O1*W*—H1*WB*···O3^ii^	0.85	2.37	3.17 (2)	158
C13—H13*A*···O2^iii^	0.93	2.67	3.570 (4)	163
C20—H20*A*···O1^iii^	0.93	2.65	3.552 (4)	164

Symmetry codes: (i) −*x*+1, −*y*, −*z*; (ii) *x*−1, *y*, *z*+1; (iii) −*x*, −*y*, −*z*+1.
